# Comparison of Short and Long-Term Outcomes of Metabolic and Bariatric Surgery in Adolescents and Adults

**DOI:** 10.3389/fendo.2020.00157

**Published:** 2020-03-24

**Authors:** Fatima Cody Stanford, Tasnim Mushannen, Priscilla Cortez, Karen J. Campoverde Reyes, Hang Lee, Denise W. Gee, Janey S. Pratt, Paul A. Boepple, Miriam A. Bredella, Madhusmita Misra, Vibha Singhal

**Affiliations:** ^1^Neuroendocrine Unit, Massachusetts General Hospital and Harvard Medical School, Boston, MA, United States; ^2^Pediatric Endocrinology, Massachusetts General Hospital and Harvard Medical School, Boston, MA, United States; ^3^MGH Weight Center, Massachusetts General Hospital, Boston, MA, United States; ^4^Weill Cornell Medicine, Education City, Al Rayyan, Qatar; ^5^Department of Biology, College of Science, University of Arizona, Tucson, AZ, United States; ^6^Liver Research Center, Beth Israel Deaconess Medical Center, Boston, MA, United States; ^7^Departments of Biostatistics and Harvard Medical School, Boston, MA, United States; ^8^Department of Surgery and Pediatric Surgery and Stanford University School of Medicine, Stanford, CA, United States; ^9^Department of Radiology, Massachusetts General Hospital and Harvard Medical School, Boston, MA, United States

**Keywords:** obesity surgery, weight loss, obesity complications, adolescent, adult, bariatric surgery

## Abstract

**Objective:** We sought to compare the short and long-term outcomes of MBS in adolescents vs. adults who have undergone a Roux-en-Y gastric bypass (RYGB) or Sleeve gastrectomy (SG).

**Design:** Retrospective cohort study.

**Setting:** Single tertiary care academic referral center.

**Participants:** One hundred fifty adolescent (≤ 21-years) and adult (>21-years) subjects with severe obesity between 15 and 70 years of age who underwent RYGB or SG.

**Outcomes:** Metabolic parameters, weight and height measures were obtained pre-and post-surgery (at 3 and 6 months, and then annually for 4 years).

**Results:** Median pre-surgical body mass index (BMI) was higher in adolescents (*n* = 76) vs. adults (*n* = 74): 50 (45–57) vs. 44 (40–51) kg/m^2^ (*p* < 0.0001). However, obesity related complications were greater in adults vs. adolescents: 66 vs. 21% had hypertension, 68 vs. 28% had dyslipidemia, and 42 vs. 21% had type 2 diabetes mellitus (all *p* < 0.010). % BMI reduction and % weight loss (WL) were greater in adolescents vs. adults at all time points (*p* < 0.050). %WL was higher in adolescents who underwent SG at each time point (*p* < 0.050), and trended higher among adolescents who underwent RYGB (*p* = 0.060), compared to adults with the respective procedure. Follow-up data showed greater resolution of type 2 diabetes and hypertension in adolescents than adults (87.5 vs. 54.8%; *p* = 0.04, and 68.7 vs. 35.4%; *p* = 0.040).

**Conclusion:** Adolescents compared to adults had greater reductions in BMI and weight, even at 4 years, and greater resolution of type 2 diabetes and hypertension. Earlier intervention in the treatment of severe obesity with MBS may lead to better outcomes.

## Introduction

Obesity, defined as a body mass index (BMI) ≥ 30 kg/m^2^ in adults and BMI ≥ 95th percentile in children and adolescents of the same age and sex, is a pandemic complex multifactorial chronic disease. Despite public health efforts to reduce obesity, its prevalence has increased to 40% in adults and 19% in youth in the United States (1–3). Obesity causes significant physical, mental, and psychosocial complications and is associated with complications like hypertension (HTN), dyslipidemia (DYS), non-alcoholic fatty liver disease (NAFLD), diabetes mellitus type 2 (T2D), obstructive sleep apnea (OSA), anxiety, and depression (4–8).

While lifestyle modifications are the cornerstones of weight management, these are usually not enough to treat obesity, especially severe obesity (BMI ≥ 40 kg/m^2^) (9). Only one weight loss medication, orlistat, is FDA approved in children and contributes to ~3% total body weight loss, insufficient in severe obesity. Timely metabolic and bariatric surgery (MBS) results in prolonged weight loss in both adolescents and adults (5, 6, 10–14). Biochemical markers of DYS, T2D, and non-alcoholic steatohepatitis (NASH) improve or resolve in most adult and adolescent patients after MBS (6, 12, 14–16). A recent study showed greater resolution of T2D and HTN in adolescents as compared to adults after Roux-en-Y gastric bypass (RYGB) suggesting a benefit of early intervention (17). However, data comparing long-term outcomes following sleeve gastrectomy (SG), the most commonly performed MBS is not available (18). Moreover, data comparing outcomes of RYGB and SG across age are lacking.

Our study seeks to compare outcomes from both RYGB and SG in a cohort of patients across a wide age spectrum at a single center. Our objective was to ascertain weight outcomes and resolution of obesity associated complications in adults vs. adolescents who undergo MBS via RYGB or SG. We hypothesized that adolescents would have better outcomes secondary to earlier intervention and overall lower disease burden at the time of intervention.

## Methods

We conducted a retrospective review of adults (> 21 years) and adolescents (≤ 21 years) with obesity (BMI ≥ 30) who underwent RYGB or SG between 2001 and 2014 at a single institution. All consecutive adolescent (*n* = 76) and adult (*n* = 74) patients were included retrospectively from 2014. We collected demographics, weight, medications, complications pre-surgery and following surgery at 3, 6, and then yearly for 4 years. Since at our center at this time adolescents were recommended surgery only after completion of linear growth, we have used the single height measurement before surgery for all subsequent time points. We do not expect adults to change their height over time at this stage of life. The clinical and laboratory parameters included in this study were systolic and diastolic blood pressures, hemoglobin A1C (HbA1c), lipids (triglycerides, LDL, HDL, total cholesterol), liver enzymes [aspartate aminotransferase (AST), alanine-aminotransferase (ALT)], iron and vitamin studies [iron, ferritin, total iron binding capacity (TIBC), vitamin B12] and 25-hydroxy vitamin D (25-OHD) levels. Time to reach the lowest weight, the lowest weight (nadir weight), and percent of the total body weight lost (TBWL) were recorded. We extracted information from patient files regarding the specific obesity related diseases, i.e., HTN, T2D, DYS, NAFLD, OSA, and PCOS (based on predetermined criteria in Supplementary Table 1). Liver biopsies were taken at the time of surgery and analyzed for liver steatosis, NASH, and/or fibrosis.

Pre-surgery data were obtained from visits within 1 year of the date of surgery. Data from the visit closest to surgery were used. Post-surgery data were collected from visits specified by time point with the given timeframes: 3 months ± 1 month, 6 months ± 1 month, 1 year ± 3 months, 2 years ± 3 months, 3 years ± 6 months, 4 years ± 6 months.

Participants who were loss to follow-up did not differ from those in whom we had follow-up data for baseline weight, gender, type of surgery, and presence of comorbidities. We have listed the subjects with follow up weight data in the table with results. For or major comorbidity assessments; type 2 diabetes mellitus (T2D)—we had follow-up data on all 31 adults and 16 adolescents who had T2D at baseline. For HTN, we had follow-up data on 48 adults (1 missing) and all 16 adolescents who had hypertension before surgery. Similarly, for dyslipidemia, we had follow-up data on 49 adults (1 missing) and all 21 adolescents who initially had dyslipidemia.

We used JMP (v13; SAS Institute, Inc. Cary, NC, USA) for analyses. Normally distributed data were analyzed using a one-way Analysis of Variance (ANOVA), and non-parametrically distributed data were analyzed using the Wilcoxon rank-sum test. Data are reported as mean ± SD for parametric values or median (IQR) for non-parametric values. Pearson's Chi Square and Fisher's Exact Test were used to compare proportions. To control for confounders, we ran linear regression models for continuous outcomes (BMI, percent weight loss) and logistic regression for nominal variables like resolution of comorbidities. We controlled for baseline weight, type of surgery, gender, race, and baseline comorbidity depending on what was relevant for consideration as a confounder. We have elaborated the rationale of using the particular confounders further with the results.

Our institutional review board—The Partners Humans Research Committee (PHRC), affiliated to Massachusetts General Hospital and Harvard Medical School approved this study.

## Results

### Baseline Characteristics

Baseline characteristics are shown in Table 1. Consistent with national trends regarding MBS, females comprised the majority in both age groups—adults and adolescents. Within both groups (adults and adolescents), the largest proportion of participants were Caucasian, followed by Hispanics, African Americans, and Asians. There was a wide distribution of ages between 15 and 70 years. Notably, the pre-surgery weight and BMI of adolescents was greater than those of adults. While both adolescents and adults were more likely to undergo RYGB vs. SG (given existing practice at that time), there was a higher prevalence of RYGB in adolescents.

**Table 1:** Baseline demographics of the adult and adolescent groups.

	**Adults (*n* = 74)**	**Adolescents (*n* = 76)**	***P***
Age	46.5 (38.0–59.3)	18.0 (17.3–19.8)	<0.010^*^
Sex (male/female)	31/43	13/63	**<0.010**
Height (in)	66.9 ± 4.2	65.9 ± 3.1	0.080
Weight (Kg)	127.3 (112.6–151.4)	140.9 (123.5–158.8)	<0.010^*^
BMI (Kg/m2)	43.6 (40.0–51.3)	50.5 (45.3–56.7)	<0.010^*^
RYGB (%)	59.5	77.6	**0.010**
SG (%)	40.5	22.4	
**Race/Ethnicity (%)**			
White	79.7	59.2	0.080
Hispanic	12.2	22.4	
African-American	2.7	7.9	
Asian	0	3.4	
>1 race	2.7	5.3	
Unknown	2.7	1.3	
Systolic BP (mm Hg)	129 (118–142)	126 (117–134)	0.220^*^
Diastolic BP (mm Hg)	77 (70.75–82)	75 (66–83.5)	0.560^*^
HDL (mg/dL)	41.5 (36.8–53.0)	39 (33.5–45.5)	0.100^*^
LDL (mg/dL)	108.6 ± 36.5	95.9 ± 4.0	**0.030**
Triglycerides (mg/dL)	146.5 (99.3–207.8)	102 (74.5–141.0)	<0.001^*^
Cholesterol (mg/dL)	184.5 (159.3–205.5)	156.0 (140.0–179.0)	<0.0001^*^
Hgb A1c (%)	6.2 (5.63–7.15)	5.6 (5.2–5.9)	<0.0001^*^
ALT (IU/L)	26.0 (16.3–36.0)	23.0 (17.0–36.0)	0.760^*^
AST (IU/L)	23.0 (17.3–29.5)	22.0 (18.0–27.0)	0.640^*^
Alkaline phosphatase (IU/L)	74.0 (59.8–87.5)	82.5 (66.0–102.0)	0.010^*^
TIBC (μg/dL)	321.6 ± 53.3	353.8 ± 61.9	**<0.010**
Iron (μg/dL)	67.0 (54.3–86.5)	54.5 (36.3–85.5)	0.020^*^
Ferritin (ng/mL)	85.0 (39.0–168.8)	56.0 (34.5–81.0)	0.020^*^
Vitamin B12 (pg/mL)	476.5 (349.3–673.5)	508.0 (396.0–620.0)	1.000^*^
Vitamin D (ng/ml)	25.5 (19.0–31.0)	23.0 (18.0–28.0)	0.210^*^

* *Wilcoxon Test was used for non-normally distributed data*.

*TIBC, Total iron binding capacity*.

*Bold value means statistically significant*.

Table 2 shows participants with pre-surgical obesity related conditions in adolescents and adults. As expected, more adults had HTN, T2D, and DYS than adolescents at the time of surgery. However, similar proportions of adolescents and adults (>80% in each) had NAFLD. More adolescents than adults had PCOS documented.

**Table 2:** Distribution of pre-surgery weight related complications.

**Adults (*n* = 74)**	**Adolescents (*n* = 76)**	***P***
Hypertension (*n* = 74): 49/74 (66.22%)	Hypertension (*n* = 76): 16/76 (21.05%)	**<0.0001**
Dyslipidemia (*n* = 74): 50/74 (67.57%)	Dyslipidemia (*n* = 76): 21/76 (27.63%)	**<0.0001**
Type 2 Diabetes Mellitus (*n* = 74): 31/74 (41.89%)	Type 2 Diabetes Mellitus (*n* = 76): 16/76 (21.05%)	**0.005**
Obstructive Sleep Apnea (*n* = 41):	Obstructive Sleep Apnea (*n* = 42)	0.845
Y: 36/41 (87.80%)	Y: 35/42 (83.33%)	
N: 5/41 (12.20%)	N: 7/42 (16.67%)	
**Non-alcoholic fatty liver disease (*****n*** **=** **69):**	**Non-alcoholic fatty liver disease (*****n*** **=** **74)**	0.085
Y: 57/69 (82.61%)	Y: 68/74 (91.89%)	
N: 12/69 (17.39%)	N: 6/74 (8.11%)	
**Steatosis (*****n*** **=** **69):**	**Steatosis (*****n*** **=** **74):**	0.091
Y: 55/69 (79.71%)	Y: 67/74 (90.54%)	
N: 14/69 (20.29%)	N: 7/74 (9.46%)	
**NASH (*****n*** **=** **69):**	**NASH (*****n*** **=** **71):**	0.953
Y: 27/69 (39.13%)	Y: 26/71 (36.62%)	
N: 42/69 (60.87%)	N: 45/71 (63.38%)	
**Fibrosis (*****n*** **=** **65):**	**Fibrosis (*****n*** **=** **71):**	0.305
Y: 32/66 (48.48%)	Y: 27/71 (38.03%)	
N: 34/66 (51.52%)	N: 44/71 (61.97%)	

*NASH, non-alcoholic steatohepatitis. Bold value means statistically significant*.

### Weight Loss

**Figure 1:**
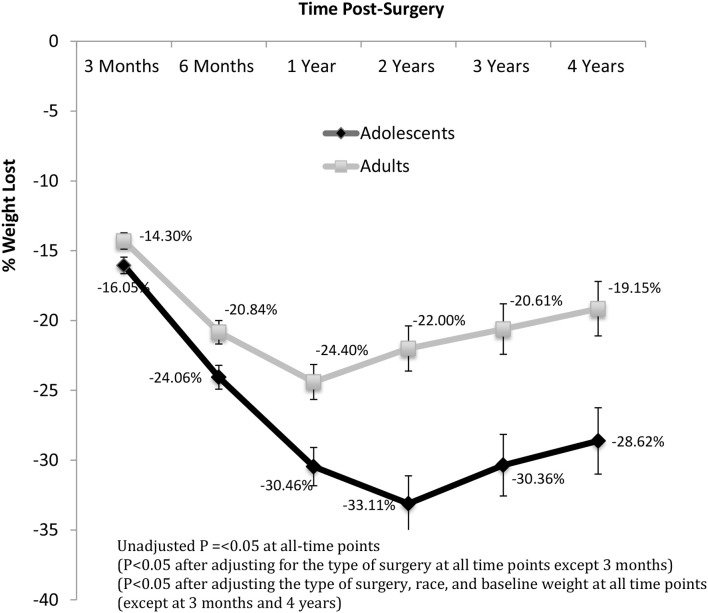
Percent of total body weight loss post-surgery over time.

Although adolescents on average had higher presurgical weight and BMI than adults, they lost a greater % of their weight than adults at every time point post-surgery (Figure 1 and Supplementary Table 1). The peak difference in %WL was reached at 2 years, at which time adolescents had lost 1.51 times more weight than adults. The percent reduction in BMI was greater in adolescents vs. adults at all time points (Data not shown—similar to the percent weight loss data as a single height measurement at the presurgical visit was used for follow up). While BMI of adolescents was higher in the early postoperative period, the difference was no longer significant at 1 year and onwards. When we controlled for the type of surgery, adolescents had more %WL at most time points (except acutely at 3 months) (Figure 1). When we controlled for surgery type, race, and baseline weight, adolescents had more % WL at most time points (except acutely at 3 months and long-term at 4 years) (Figure 1). Inge and colleagues demonstrated similar %WL response at 5 years after RYGB, but their cohort did not include patients who underwent SG, (17) a procedure which has less %WL in the midterm and long-term based upon recent meta-analysis and systematic reviews (19). It is also important to note that our cohort likely differs from national data as our center is a tertiary referral center with patients with a high level of medical complexity.

Adolescents also showed greater %WL when they were stratified by surgical technique. At every year post-surgery, adolescents who underwent SG had greater %WL than adults (*p* < 0.05) (Table 3). These differences in %WL persisted after controlling for pre-surgical weight, race and gender. Specifically, after 4 years of SG, adolescents had lost 25–29% of their weight, while adults had lost 14–21% of their presurgical weight. However, %WL following RYGB did not differ in adolescents vs. adults at any time point. Adults who underwent RYGB vs. SG had greater %WL at all timepoints (even after controlling for baseline weight). This superiority of RYGB over SG was not observed in adolescents in our cohort.

**Table 3:** Percent weight loss at different time points stratified by surgery type.

	**Adults**		**Adolescents**		***P***			
	**RYGB (*n* = 44)**	**SG (*n* = 30)**	**RYGB (*n* = 59)**	**SG (*n* = 17)**	**RYGB vs. SG (Adult)**	**RYGB vs. SG (Adol)**	**RYGB Adult vs. Adol**	**SG Adult vs. Adol**
3 Months	−15.9 ± 4.8 (*n* = 44)	−12.0 ± 4.8 (*n* = 30)	−16.0 ± 4.8 (*n* = 55)	−16.3 ± 5.3 (*n* = 17)	**0.0008**	0.843	0.939	**0.006**
6 Months	−23.1 ± 6.7 (*n* = 37)	−17.2 ± 7.8 (*n* = 23)	−24.4 ± 5.1 (*n* = 46)	−22.9 ± 4.9 (*n* = 12)	**0.002**	0.374	0.341	**0.027**
1 Year	−26.7 ± 10.5 (*n* = 36)	−21.1 ± 9.9 (*n* = 25)	−30.7 ± 8.9 (*n* = 43)	−29.2 ± 8.5 (*n* = 8)	**0.039**	0.670	0.071	**0.045**
2 Years	−30.2 (−36.2 to 20.5) (*n* = 31)	−19.3 (−26.7 to 12.5) (*n* = 25)	−33.8 (−40.6 to 26.9) (*n* =30)	−24.9 (−41.8 to 22.7) (*n* = 7)	0.022^*^	0.473^*^	0.059^*^	**0.028**
3 Years	−26.5 ± 10.9 (*n* = 30)	−13.6 ± 12.3 (*n* = 25)	−31.0 ± 14.1 (*n* = 30)	−27.9 ± 13.2 (*n* = 7)	**0.0001**	0.601	0.172	**0.011**
4 Years	−24.7 ± 11.2 (*n* = 29)	−11.2 ± 12.3 (*n* = 20)	−32.3 (−36.6 to 22.5) (*n* = 26)	−24.4 (−27.4 to 19.1) (*n* = 7)	**0.0002**	0.243^*^	0.135^*^	0.011^*^

* *Wilcoxon Test used for non-normally distributed data. Bold value means statistically significant*.

Despite a dramatic response to surgery in both cohorts, a substantial proportion of patients continued to have obesity with a BMI ≥ 30 kg/m^2^ (69.3% in adolescents vs. 62.2%, in adults; *p* = 0.4) and BMI ≥ 35 kg/m^2^ (46.7% in adolescents vs. 35.1% in adults, *p* = 0.15) at nadir. At 4 years follow-up, 75.8% of adolescents vs. 81.6% of adults had a BMI ≥ 30 kg/m^2^ (*p* = 0.02), and 60.6% of adolescents vs. 53.1%, of adults had a BMI ≥ 35 kg/m^2^ (*p* = 0.02).

### Resolutions and Improvements

#### Hypertension

**Figure 2:**
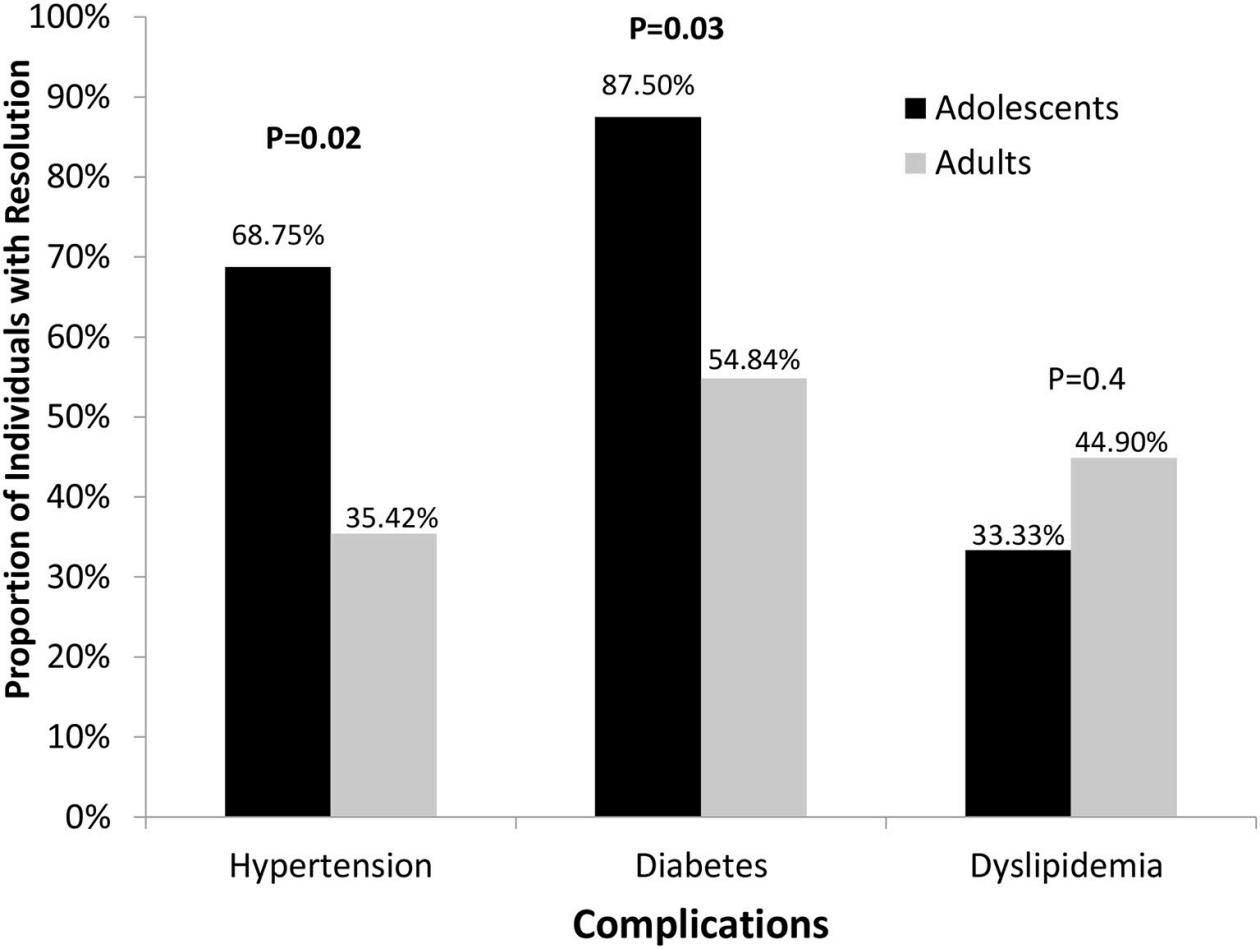
Proportion of subjects with resolution of hypertension, diabetes, and dyslipidemia.

Among participants pre-surgically diagnosed with HTN and who had follow-up data (*n* = 64; no data in 1 adult patient), almost two times as many adolescents vs. adults attained resolution of HTN and were off the medications at some time point over the course of four years (*p* = 0.02 unadjusted, *p* = 0.05 after adjusting for type of surgery) (Figure 2). Among all participants with follow-up data, regardless of a pre-surgical diagnosis of HTN and antihypertensive medication use, the proportion of participants with a blood pressure ≥ 140 mm systolic or ≥ 90 mm diastolic was similar between adults and adolescents at all time points post-surgery except at 1 year, at which time only 2.6% adolescents vs. 20.9% adults had documented blood pressures above 140/90 mm Hg (*p* = 0.015). Adolescents had either similar or better blood pressures than adults and were more likely to have resolution of HTN.

#### Type 2 Diabetes Mellitus

87.5% adolescents (*n* = 16) vs. 54.8% adults (*n* = 31) had resolution of T2D (*p* = 0.03) over the course of 4 year follow up (Figure 2). Because the pre-surgical HbA1C and the type of surgery may affect the resolution of T2D, we consider them as confounder and controlled for these variables in our analyses. Adolescents had significantly lower HbA1C levels than adults at 3 months (5.39 vs. 5.83%; *p* = 0.002) and 4 years (5.29 vs. 6.27%; *p* = 0.02). A similar pattern was observed for changes in HbA1C values from baseline after controlling for the same variables, with greater reductions observed in adolescents than in adults at 3 months (−1.14 vs. −0.70%) and 4 years (−1.46 vs. −0.57%) (*p* < 0.02 for both).

#### Dyslipidemia

Adolescents did not differ from adults for the proportion that had resolution of dyslipidemia or change in absolute values of triglycerides, LDL, or total cholesterol at any time point post-surgery (Figure 2). However, after controlling for baseline HDL, surgery type, and gender, adults had higher HDL levels than adolescents in the early post-operative period at 3- and 6-months post-surgery (43.8 vs. 39.6 mg/dl, *p* = 0.018; 49.1 vs. 43.1 mg/dl, *p* = 0.032, respectively). However, subsequently, this difference was no longer observed, and HDL levels of adolescents rose to values similar to those observed in adults (*p* > 0.3). From 1 to 4 years, HDL values in adults ranged between 53 and 61 mg/dl while those of adolescents ranged between 55 and 58 mg/dl. By 1-year post-op, adolescents experienced substantially larger increases in HDL than adults.

#### Obstructive Sleep Apnea (OSA)

Assessments of resolution of OSA were not optimal. Approximately 95% of adults and 89% of adolescents who had OSA pre-surgically did not undergo a sleep study following surgery. However, the majority who did OSA had documented improvements of OSA noted in their medical records. There was no difference in the proportions of adolescents vs. adults with documented improvements of OSA at all time points except at 6 months, when 81.3% of adolescents vs. 45.0% of adults had documented improvement (*p* = 0.04). After 6 months, the proportion of adolescents with OSA improvement remained steady between 88 and 94%, while that in adults rose from 45% at 6 months to 75% at 4 years.

#### NAFLD and Liver Enzymes

Post-surgical assessments for NAFLD were rarely done as most patients did not undergo liver biopsies after surgery. Post-surgical information was only available in 24/76 (32%) of adolescents and 25/75 (33%) of adults; nevertheless, based on our predetermined definitions of NAFLD resolution (normalization of liver biopsy and/or liver enzymes if previously elevated), 16/24 (66.7%) of adolescents and 21/25 (84.0%) of adults attained resolution (*p* = 0.16).

ALT and AST levels in adolescents and adults were similar pre-surgically (23 vs. 26 U/L, *p* = 0.76; and 22 vs. 23 U/L, *p* = 0.64, respectively). At 3 months post-surgery, both ALT and AST levels were significantly higher in adolescents vs. adults (29.0 vs. 21.5 U/L, *p* = 0.03; and 26.0 vs. 22.5 U/L, *p* = 0.03, respectively). By 4 years post-surgery, ALT and AST levels were not different between adolescents and adults (23.0 vs. 19.7 U/L, *p* = 0.43; and 26.1 vs. 22.5 U/L, *p* = 0.19, respectively).

#### Vitamin and Mineral Deficiencies

25(OH)-D levels were similar in adolescents vs. adults pre-surgery (Table 1) and they were similar at the 4-year follow up (24 ± 12 vs. 29 ± 12 ng/dl; *p* = 0.22).

Ferritin and iron levels were higher in adults vs. adolescents pre-surgery (Table 1). At 4 years, ferritin and iron levels were similar in adolescents vs. adults [14.5 ng/mL (5.5–33.7) vs. 13 ng/mL (9.5–44.5); *p* = 0.22 and 60.5 μg/dl ± 29 vs. 75.1 μg/dl ± 43; *p* = 0.44, respectively].

Although adolescent and adult vitamin B12 levels were similar pre-surgically (Table 1), they were consistently lower in adolescents 4-years following surgery (485.4 ± 339 vs. 1027 ± 645, pg/mL respectively; *p* = 0.003).

## Discussion

Our study demonstrates that early intervention with MBS during adolescence leads to better weight loss outcomes and obesity related disease resolution compared to MBS performed in adulthood. To our knowledge, this is the first study to evaluate MBS outcomes after both RYGB and SG in adolescents and adults.

Literature comparing short and long-term outcomes of MBS (RYGB and SG) in adolescent and adult populations is scarce, and our data are promising as they demonstrate that outcomes are as better in adolescents compared to adults, especially for SG. The Swedish Obese Study (SOS) reviewed 5-year-follow-up data in adolescents vs. adults who underwent RYGB, and reported a mean reduction in BMI of 13.1 kg/m^2^ in adolescents vs. 12.3 kg/m^2^ in adults after 5 years of MBS (7). Khidir et al. showed a greater decrease in %WL after SG in adolescents vs. adults at 5-year follow-up (20). Similarly, Benedix et al. found that adolescents who underwent SG had better weight loss outcomes compared to adults. They described higher rates of resolution of HTN, T2D, and OSA in adolescents (21). However, these studies did not perform a head-to-head comparison of outcomes in different types of MBS, particularly RYGB vs. SG, the two most commonly performed MBS procedures in the adolescent and adult population. To our knowledge, this is one of the first studies to highlight the greater efficacy of MBS in adolescents compared to adults 4 years after SG or RYGB.

In our study, adolescents had better pre-surgery lipid, liver, glycemic, and hematological parameters, but their BMI was higher than adults. By the time of MBS, ~1/5 of adolescents had at least one obesity related complication, which over time increases their risk of mortality and adversely affects their quality of life (21). It is thus important to identify adolescents with moderate to severe obesity and escalate their obesity treatment according to the severity of their obesity.

Our data demonstrate that adolescents have greater weight loss than adults both acutely and at 4-year follow-up following MBS. Despite starting at a higher BMI than adults, the nadir BMI after MBS was not different between adults and adolescents suggesting that MBS has greater weight loss benefits at a younger age. Despite these positive results, almost 2/3 of the adolescents continued to have obesity at nadir weight and almost 50% had severe obesity. This is likely because adolescents often have delayed MBS intervention and have greater severity of obesity than adults at the time of MBS.

Studies have shown variation with regards to the ideal procedure in adults and adolescents (i.e., RYGB vs. SG). This study shows that regardless of the surgical technique utilized, adolescents have greater reductions in total body weight than adults over both the short and long-term. In our cohort, adults showed greater weight loss with RYGB than with SG at all time points. This superiority of RYGB was not seen in the adolescent group. Adolescents had a better response to SG than adults, which is consistent with previous studies (22, 23). This is encouraging and supportive of the current trend of more frequent SG, with its reduced complexity and lower post-surgical prevalence of micronutrient deficiencies than RYGB, particularly in adolescents (18).

Adolescents showed greater resolution of obesity related complications, especially HTN and T2D, than adults. These findings are supported by other studies which show that a longer duration of diabetes before MBS reduces the chances of resolution of T2D (24). Prolonged hyperglycemia and β-cell stress may lead to irreversible pancreatic damage and decrease the chances of improvement in glucose tolerance. Similar proportions of adolescents and adults had resolution of DYS, OSA and normalization of liver transaminases. After 6 months, adolescents had lower ALT levels than adults and maintained this at 4 years. Based on documentation in the medical records, adolescents had more rapid improvements of OSA, which suggests lower severity and greater reversibility of OSA in this age group. These data show that early MBS is equally or more beneficial in resolving obesity complications.

We did not find any difference between groups for vitamin D or iron levels. However, adolescents had lower levels of vitamin B12 after surgery than adults, suggesting lower compliance with supplement intake. This emphasizes the importance of follow-up and ongoing surveillance. While micronutrient deficiencies are a challenge, their long-term clinical impact in adolescents is unknown.

### Limitations of the Study

As this was a retrospective study in one academic medical center, it might be challenging to extrapolate our results to other institutions. Despite this limitation, few centers in the US operate on both adult and pediatric patients with moderate to severe obesity, and this offers a unique opportunity to compare the two groups. There were limitations inherent to any retrospective study. Specifically, the number of participants who returned for follow up at the different timepoints was variable, and tests done at each time point were inconsistent.

We did not have enough follow up data to document resolutions of OSA and NAFLD in both adolescent and adult groups because only a small proportion had post-surgical polysomnography and liver biopsies and hence we only report documented improvements. Also, we were not able to obtain data on long term surgical complications due to inconsistent documentation. Moreover, despite having a diverse population, the predominant race was white, limiting the generalizability of these results to other racial and ethnic groups who may respond differently to MBS. Larger prospective studies that ensure racial diversity are necessary to confirm these findings.

## Conclusion

Despite higher weight and BMI of adolescents at the time of MBS, adolescents had a greater percent total body weight loss and resolution of obesity co-morbidities (when present) than adults regardless of whether they underwent RYGB or SG. Our study results suggest that MBS surgical intervention at an early age portends better outcomes with regards to weight loss and obesity co-morbidity. As such, we encourage more utilization of MBS in adolescents with moderate to severe obesity as they are the age cohort most likely to glean benefits.

## Data Availability Statement

The datasets generated for this study are available on request to the corresponding author.

## Ethics Statement

The studies involving human participants were reviewed and approved by Partners Institutional Review Board. Written informed consent from the participants' legal guardian/next of kin was not required to participate in this study in accordance with the national legislation and the institutional requirements.

## Author Contributions

KC, TM, PC, and VS were responsible for acquisition of data and data analyses. FS, VS, and KC were responsible for interpreting the data. KC, TM, and PC were responsible for drafting the manuscript. FS and VS were responsible for the conception and design of the study and critical review of the paper. FS, MM, and VS were responsible for revising the article critically and adding important intellectual content. DG, PB, JP, MB, and HL were responsible for data collection and providing critical appraisal of the content. All authors have read and approved the final version of the paper and have made substantial contribution to the paper.

### Conflict of Interest

The authors declare that the research was conducted in the absence of any commercial or financial relationships that could be construed as a potential conflict of interest.
