# Supplementary material for: Comparison of Short and Long-Term Outcomes of Metabolic and Bariatric Surgery in Adolescents and Adults
Source: Front Endocrinol (Lausanne). 2020 Mar 24;11:157. doi: 10.3389/fendo.2020.00157 (PMC7105703; doi:10.3389/fendo.2020.00157)
Supplement: Supplementary file 1 [file Data_Sheet_1.docx]

| **Comorbidity** | **Definition** | **Resolution** |
| --- | --- | --- |
| **DM2** | Medical records + HbA1c ≥ 6.5% or hypoglycemic medication | HbA1c ≤ 6.5% and off of hypoglycemic medications |
| **DYS** | Medical records + total cholesterol > 200 mg/dL, triglycerides > 150mg/dL, or HDL < 50 mg/dL in women or <40 mg/dL in men and/or lipid-lowering medication | Normalization of lipid panels and off statins |
| **HTN** | Medical records + systolic/diastolic blood pressure > 140/90 or antihypertensive medication | Normalization of systolic/diastolic blood pressure and off antihypertensives |
| **OSA** | Polysomnography study (apnea hypopnea index (AHI) ≥ 5) or mention of the results in the medical records | Post-surgery polysomnography study which demonstrated an AHI ≤5 |
| **NAFLD** | Liver biopsies showing steatosis, steatohepatitis or fibrosis  (Elevated ALT and/or AST levels secondarily confirmed NAFLD but were not required for a diagnosis of NAFLD) | Repeat liver biopsy post surgery or ALT normalization if previously high |

**Supplementary Table 1: Predetermined Definitions of Diagnosis**

**Supplemental Table 2: % Weight Loss Post-Surgery**

|  | **3 Months** | **6 Months** | **1 Year** | **2 Years** | **3 Years** | **4 Years** |
| --- | --- | --- | --- | --- | --- | --- |
| **Adult** | -14.3 ± 5.2  (n=74) | -20.8 ± 7.7  (n=60) | -24.4 ± 10.5  (n=61) | -22.0  (-34.1 to -14.5)  (n=56) | -20.6 ± 13.1  (n=55) | -19.2  (-28.6 to -9.4)  (n=49) |
| **Adolescent** | -16.1 ± 4.9  (n=72) | -24.1 ± 5.0  (n = 58) | -30.5 ± 8.8  (n=51) | -33.1  (-40.6 to -25.1)  (n=37) | -30.4 ± 13.8  (n=37) | -28.6  (-36.5 to -20.2)  (n=33) |
| **Ratio (%WL adolescents: %WL adults)** | 1.12 | 1.15 | 1.25 | 1.51 | 1.47 | 1.49 |
| **P-value** | **0.037** | **0.008** | **0.001** | **0.0009*** | **0.0009** | **0.0079*** |
